# Mad moves of the building blocks – nucleotide sugars find unexpected paths into cell walls

**DOI:** 10.1093/jxb/ery026

**Published:** 2018-02-23

**Authors:** Georg J Seifert

**Affiliations:** University of Natural Resources and Life Science, BOKU Vienna, Department of Applied Genetics and Cell Biology, Vienna, Austria

**Keywords:** Arabidopsis, cell wall, nucleotide sugar, transporter, UDP-glucose, UDP-xylose synthase, UDP-xylose, xylan

## Abstract

This article comments on:

Zhao X, Liu N, Shang N, *et al.* 2018. Three UDP-xylose transporters (UXTs) participate in xylan biosynthesis by conveying cytosolic UDP-xylose into the Golgi lumen in Arabidopsis. Journal of Experimental Botany 69, 1125–1134..

**A raft of recent studies, including a new paper by Zhao *et al.* (2018), has shed new light on the importance of cellular topology in controlling the flux of nucleotide sugars to their destinations. It is an exciting time, as the rapidly progressing elucidation of the convoluted flux of carbon bodies through nucleotide sugar metabolism and inter-organellar transport means we are approaching an understanding of the control principles of plant cell wall polysaccharide biosynthesis.**

Plant science is approaching the vision of cell walls constructed ‘fit for purpose’ (Johnson *et al.*, 2017). The assembly of carbohydrates from monosaccharides encompasses the stereospecific transfer of specific (di)phosphonucleotide-activated sugars onto specific acceptor molecules by glycosyltransferases estimated to be encoded by well over 500 Arabidopsis loci (Hansen *et al.*, 2012). At least 16 nucleotide sugars are generated in the nucleotide sugar interconversion network and many of the enzymes involved have now been cloned and characterized (Reiter and Vanzin, 2001; Seifert, 2004; Reiter, 2008; Bar-Peled and O’Neill, 2011). More recently, Golgi-localized nucleotide sugar transporters have been identified (Temple *et al.*, 2016). But how does nucleotide sugar metabolism and transport align with carbohydrate biosynthesis in the living plant?

## Regulation of substrate flux

Most nucleotide sugars are made from UDP-D-glucose (UDP-Glc) in the cytosol (Box 1). This central carbohydrate precursor is converted to UDP-L-rhamnose, UDP-galactose and UDP-D-glucuronic acid (UDP-GlcA), the latter being converted to UDP-D-apiose and UDP-D-xylose (UDP-Xyl). Only the enzyme that generates UDP-D-galacturonic acid (GalA) from UDP-GlcA is exclusively Golgi localized. By contrast, two UDP-sugar interconverting enzymes exist in two forms localized in topologically separated compartments – one form in the cytosol and another one in the Golgi. The reason for this plant-specific duality is not immediately apparent.

**Box 1. Metabolic pathways of UDP-Xyl and UDP-Ara*f***

Brown arrows indicate the fate, *in planta*, of carbon bodies ending up as cell wall-associated xylose: glucuronoxylan (GX), xyloglucan (XG), xylogalacturonan (XGA) and complex N-linked glycan (CGL). Green arrows indicate the fate of carbon bodies ending up as arabinofuranose bound to cell wall polymers: rhamnogalacturonan I (RG I) and RG II, arabinogalactan protein (AGP) and extensin (EXT). The red arrow indicates the flux from UDP-Glc to UDP-GlcA that is shared between arabinose and xylose bodies and mediated by UDP-Glc dehydrogenase (UGD). Thin grey arrows indicate potential metabolic routes that are apparently not used *in planta*. The enzymes involved are UDP-Glc dehydrogenase (UGD), UDP-Xyl synthase (UXS), UDP-xylose 4-epimerase (UXE) bispecific UDP-Glc/UDP-Xyl 4-epimerase (UGE), arabinokinase (AK), UDP-sugar pyrophosphorylase (USP), and reversibly glycosylated polypeptide (RGP). Nucleotide sugar transporters are UDP-Xyl transporter (UXT), UDP-uronic acid transporter (UUAT), a hypothetical UDP-Ara*p* export facilitator (grey), and UDP-arabinofuranose transporter (UAfT). The symbols for ‘xylosyltransferase’ (XT) and ‘arabinosyltransferase’ (AT) represent different glycosyltransferases generically. Note that pathways leading to UDP-galactose, UDP-L-rhamnose, and UDP-galacturonic acid or any GDP-sugars are not shown.

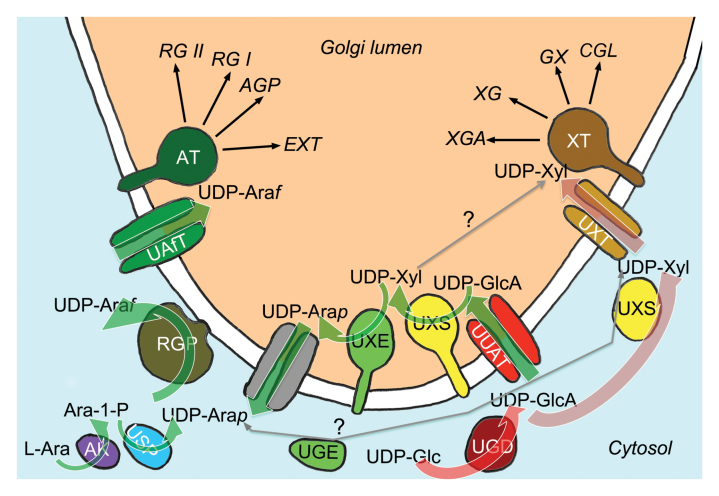

The first example is the biosynthesis of UDP-Xyl, a precursor for xylans (York and O’Neill, 2008), xyloglucan, rhamnogalacturonan II (RG II) and xylogalacturonan (Caffall and Mohnen, 2009). UDP-Xyl synthase (UXS) catalyses the decarboxylation of UDP-GlcA to UDP-Xyl with the *UXS3*, *-5* and *-6* genes encoding cytosolic UXS and the *UXS1*, *-2* and *-4* loci encoding the Golgi-localized isoforms. Because UDP-Xyl is exclusively required inside the Golgi, the three cytosolic UXS isoforms appear redundant. More crucially, to give the cytosolic pool of UDP-Xyl access to the site of carbohydrate biosynthesis, a Golgi-localized UDP-Xyl transporter is needed. Indeed, three UDP-Xyl transporters (UXT1–3) were ingeniously identified using a biochemical assay and the *uxt1* mutant showed a defect in glucuronoxylan (GX) structure and abundance. This phenotype suggested that polysaccharide biosynthesis somehow depended on cytosolic UDP-Xyl despite the presence of functional UXS in the Golgi. However, there was only a relatively minor reduction of total cell wall xylose in *uxt1* and no carbohydrate other than GX was affected in the mutant. Moreover, the *uxt2 uxt3* double mutant was phenotypically normal (Ebert *et al.*, 2015).

This key paper by Ebert *et al.* (2015) opened important questions for understanding how substrate flux is regulated *in planta*. Does the GX-specific defect in *uxt1* reflect substrate channelling from UXT1 to GX-specific xylosyltransferases? And *vice versa,* is the relatively subtle overall phenotype of *uxt1* due to genetic redundancy in UDP-xylose transport or the action of Golgi-localized UXS? Zhao *et al.* (2018) address these questions, showing that in the *uxt1 uxt2 uxt3* triple mutant, plant growth and secondary cell wall thickening are severely affected and, in addition to GAX, xyloglucan is also defective. Although not contradicting the substrate channelling hypothesis their paper conclusively demonstrates the crucial importance of the cytosol to Golgi transport of UDP-Xyl for multiple xylose-containing carbohydrates. This further corroborates previous triple-mutant studies comparing the two forms of UXS, which showed that cytosolic UXS, but not the Golgi-localized enzyme, affected the incorporation of xylose into cell wall polysaccharides (Kuang *et al.*, 2016; Zhong *et al.*, 2017).

## Mechanistic basis of separation

What might be the mechanistic basis for this apparent separation of metabolite fluxes (Box 1)? A common feature of both UXS and UDP-Glc dehydrogenase (UGD), which generates UDP-GlcA, is that both enzymes are inhibited by UDP-Xyl (Harper and Bar-Peled, 2002; Pattathil *et al.*, 2005; Klinghammer and Tenhaken, 2007). Therefore, developmental up-regulation of cytosolic UXS at the onset of secondary cell wall formation would increase cytosolic UDP-Xyl, thereby feedback-inhibiting UGD. The inhibition of UGD would down-regulate the biosynthesis of both UDP-GalA and, via Golgi-localized UDP-Xyl biosynthesis, UDP-L-arabinose (UDP-Ara), which are mainly needed during primary cell wall biosynthesis. But if xylose-containing carbohydrates receive UDP-Xyl from cytosolic UXS, what is the biological function of the Golgi form? Two genetic studies failed to establish a clear role for *UXS1, -2* and *-4* (Kuang *et al.*, 2016; Zhong *et al.*, 2017). However, as the loci are primarily expressed in tissues that undergo primary cell wall formation (Kuang *et al.*, 2016), it would be interesting to further investigate the *uxs1 uxs2 uxs4* carbohydrate-chemical phenotype in actively expanding tissues.

Indirect evidence for the importance of Golgi-localized UXS in providing the substrate for UXE and ultimately for arabinosyltransferases came from the finding that a transporter for UDP-GlcA, called UUAT1, was important for the arabinose content of cell walls (Saez-Aguayo *et al.*, 2017). Once inside the Golgi, UDP-GlcA is either converted to UDP-GalA by UDP-glucuronic acid 4-epimerase (GAE) or to UDP-Xyl by UXS. From UDP-Xyl, UDP-Xyl 4-epimerase (UXE) generates UDP-Ara and, crucially, the *uxe1* mutant showed a dramatic reduction in cell wall arabinose content (Burget and Reiter, 1999; Burget *et al.*, 2003).

## Pentagonal arabinofuranose, hexagonal pyranose

Another crucial aspect of UDP-Ara biosynthesis is that the pentagonal arabinofuranose (Ara*f*) but not the hexagonal pyranose (Ara*p*) form of L-arabinose is found in most cell wall polymers. However, a mutase is required for efficient conversion of the product of UXE UDP-Ara*p* into UDP-Ara*f*. This mutase is a protein complex encoded by the *REVERSIBLY GLYCOSYLATED POLYPEPTIDE1*, *-2* and -*5* (*RGP1, -2* and *-5*) loci and is partially bound to the cytosolic face of the Golgi. Knockdown of *RGP1* and *RGP2* resulted in massive growth defects and a near-complete loss of cell wall arabinose (Rautengarten *et al.*, 2011). The recently identified UDP-arabinofuranose transporter UAfT (Rautengarten *et al.*, 2017) channels UDP-Ara*f* back into the Golgi where it is the substrate for multiple arabinosyltransferases. However, the potential metabolite pathways into cell wall-linked Ara*f* do not end here.

UDP-Ara*p* exported from the Golgi to the cytosol is expected to mix with UDP-Ara*p* generated from free L-arabinose during carbohydrate breakdown using arabinokinase and UDP-sugar pyrophosphorylase (Geserick and Tenhaken, 2013; Behmüller *et al.*, 2016). This salvage pathway is the mechanistic explanation for the reversion of the *uxe1* phenotype by experimentally applied L-Ara (Burget and Reiter, 1999). And yet another pathway potentially leads to UDP-Ara*f.* The cytosolic bifunctional UXE/UDP-glucose 4-epimerase (the UGE1 and UGE3 isoforms) (Kotake *et al.*, 2009) should in fact generate cytosolic UDP-Ara*p* from the plentiful supply of UDP-Xyl by cytosolic UXS. This potential option, however, is contradicted by the strong arabinose deficiency in cell walls in the *uxe1* mutant (Burget and Reiter, 1999) and the absence of a phenotype in the *uge1 uge3* double mutant (Rösti *et al.*, 2007). One possible explanation might be that precise co-expression at the level of both mRNA and protein is required for normal metabolite flux during growth and development. Another possibility to explain the observed specificity is substrate channelling between adjacent enzymes and transporters assembled in a complex. At any rate, while the metabolic pathways of nucleotide sugars appear capricious, they undoubtedly represent a flexible and efficient delivery system hardened by billions of years of evolution.
